# Advances in Indoor Positioning and Indoor Navigation

**DOI:** 10.3390/s22197375

**Published:** 2022-09-28

**Authors:** Antoni Perez-Navarro, Raúl Montoliu, Joaquín Torres-Sospedra

**Affiliations:** 1 Faculty of Computer Science, Multimedia and Telecommunication, Universitat Oberta de Catalunya (UOC), Rambla del Poblenou, 156, 08018 Barcelona, Spain; 2 Institute of New Imaging Technologies, Universitat Jaume I, 12071 Castellón, Spain; 3 ALGORITMI Research Centre, Universidade do Minho, 4800-058 Guimarães, Portugal

## 1. Contributions to the Special Issue

Locating devices in indoor environments has become a key issue for many emerging location-based applications and intelligent spaces in different fields. However, there is presently no overall and easy solution. Despite a large-scale deployment of such location systems is not available yet, this strategic topic is called to lead technological innovations of great impact on the daily activities of people in the coming years, in areas such as health and independent living, leisure, security, etc.

However, applications deal with very different scenarios. For example, Jansen et al. [1] deal with challenging scenarios in indoor navigation that include rain, fog, dust, or dirt. Their system is based on single or several sonar sensors using a layered control system. The main novelty of their work is that sensors can be freely positioned in mobile devices. On the other hand, Ninnemann et al. [2] propose the deployment of sensors to achieve a multi-purpose connected aircraft cabin able to give, among other services, passenger localization and occupancy detection. They use a multipath-assisted radio sensing (MARS) approach using the propagation information of transmitted signals, which are provided by the channel impulse response (CIR) of the wireless communication channel.

In this Special Issue, we find works that deal with the more cutting-edge elements in indoor positioning. We see that Ultra Wide Band (UWB) is increasing its role. However, inertial systems are still a common mechanism of positioning, with dedicated devices as inertial measurement units (IMUs) but also using the smartphone as an IMU. The problem with these systems is the drift with movement, and authors have looked for several mechanisms to compensate for that drift, such as using magnetic fields or artificial intelligence (AI).

The difficulties of using one mechanism or another can be partially solved by using different systems fused, which is called fusion systems. In these systems, AI plays again an important role. AI, in fact, plays an important role in indoor positioning, and it appears explicitly or implicitly in many location systems.

A very promising mechanism of positioning is based on light. They usually need very specific devices but offer very accurate systems in controlled environments.

Finally, one of the big challenges that indoor positioning faces is to compare and evaluate systems. In the current Special Issue, several papers are devoted to this end.

In the following sections, a short note of the papers in the Special Issue about these items is shown.

### 1.1. Ultra Wide Band

Mascher et al. [3] deal with a stressful situation: the location of military troops in environments without GNSS available, which is called blueforce tracking. They propose a system, NIKE BLUETRACK, that combines a dual foot-mounted inertial system, with opportunistic UWB and a 3D tunnel model, using a particle filter. They achieve positioning errors of 1 m.

Another challenging scenario is robot localization, for example, in Bae et al. [4], since they usually need high accuracy. Once again, we find that UWB can compensate bias.

Regarding UWB, the standard IEEE 802.15.4z was recently released to increase both the robustness and the security of the underlying message exchanges. Most of the work is theoretical, but Tiemann et al. [5] show an empirical evaluation of the ranging performance in realistic environments and also assess the robustness to different sources of interference.

### 1.2. Magnetic Field and Inertial Systems

Viset et al. [6] also use an inertial system for positioning and dead reckoning. In this case, to compensate for the drift of these systems, they use an Extended Kalman Filter (EKF) for magnetic field for simultaneous localization and mapping (SLAM).

Harder et al. [7] also use an IMU but embedded in the smartphone. They propose a novel real-time map-matching approach that was developed using a backtracking particle filter, which reduces complexity and improves flexibility. Authors achieve 3 m of accuracy at approximately the 90th percentile.

The magnetic field is increasing its importance as a positioning tool. However, it has some instabilities and device dependence that can make it unreliable in some cases. Ouyang et al. [8] deal with the features of the magnetic field. They propose a mechanism to eliminate the anomalies and test the localization performance of heterogeneous smartphones. Finally, they summarize the feasibility/limitations of using only magnetic field measurement for indoor localization

### 1.3. Artificial Intelligence and Fusion Systems

AI is nowadays being applied in many situations, and indoor positioning is one of them. Koutris et al. [9] propose a deep-neural-network-based indoor localization method. They use the received signal strength indicator (RSSI) and the in-phase and quadrature-phase (IQ) from Bluetooth Low-Energy (BLE) Anchor Points (APs) to estimate the angle of arrival (AoA) at all APs. They obtain a precision of 70 cm.

It is important to note that methods to obtain indoor positioning are not isolated and can be fused. This is what Kia et al. [10] propose in their work, where they combine UWB and WiFi RSSI. They use WiFi Round-Trip Time (RTT) and UWB Two-Way Ranging (TWR) methods and obtain the result that UWB and WiFi compensate each other’s limitations.

Neunteufel et al. [11] deal with the problem of multipath faces for radio signals, which affect methods based on received signal strength (RSS), angle of arrival (AoA), or time of flight (ToF) measurements. In this work, they present a measurement campaign using a receiver infrastructure based on software-defined radio (SDR) platforms. They analyze the effects of possible anchor placement schemes and scenario geometries and achieve an error of 2.19 m.

### 1.4. Light Based Systems

De La Llana-Calvo et al. [12] face the problem of orientation. Their approach is based on position-sensitive device (PSD) sensors and the visible light emitted from the illumination of the room in which it is located. The authors achieved a precision of 0.2${}^{\circ }$ for two emitters. On the other hand, by using four light sources, the three Euler rotation angles were determined, with mean errors in the measurements smaller than 0.35${}^{\circ }$ for the x- and y-axes and 0.16${}^{\circ }$ for the z-axis.

Light is also used by Aparicio Esteve et al. [13]. They propose an indoor local positioning system based on LED lighting, transmitted from a set of beacons to a receiver. The receiver is based on a quadrant photodiode angular diversity aperture (QADA) plus an aperture placed over it. They use the perspective-n-point (PnP) problem the and IPPE algorithm. They obtain similar results to those obtained with triangulation techniques, but with this new algorithm, they were also able to obtain the *z* component.

### 1.5. Location Systems Evaluation and Comparison

Localization error is a common metric given in indoor positioning work. However, how reliable are these errors? This is the question that Anagnostopoulos et al. [14] ask. They present an open-source, reproducible benchmarking framework for evaluating and consistently comparing various methods of Dynamic Accuracy Estimation (DAE). They evaluate multiple methods of DAE using open data, open code, and a rich set of relevant evaluation metrics.

Schyga et al. [15] also deal with the evaluation problem and the difficulty to compare results among different sources. They propose the T&E 4iLoc Framework, a methodology for T&E of indoor localization systems in semi-controlled environments based on a system-level and black-box approach, which is highly reproducible and, therefore, facilitates comparison.

To compare Ultrasonic Indoor Positioning Systems (UIPSs), Pérez-Rubio et al. [16] present the CODEUS platform, which includes a simulator tool and an online experimental demonstrator. CODEUS provides results and performance analysis for different metrics and at different stages of signal processing.

Ren et al. [17] compare several systems but focus on a very specific collective: people who are blind. Authors compare several algorithms using inertial sensors for pedestrian tracking, as applied to data from WeAllWalk, the only published inertial sensor dataset collected indoors from blind walkers. Their results show the importance of testing algorithms with data from this collective.
